# Dissection of the molecular targets and signaling pathways of Guzhi Zengsheng Zhitongwan based on the analysis of serum proteomics

**DOI:** 10.1186/s13020-019-0252-y

**Published:** 2019-08-27

**Authors:** Baojin Yao, Jia Liu, Duoduo Xu, Daian Pan, Mei Zhang, Daqing Zhao, Xiangyang Leng

**Affiliations:** 1 Jilin Ginseng Academy, Changchun University of Chinese Medicine, Changchun, 130117 Jilin China; 2 College of Pharmacy, Changchun University of Chinese Medicine, Changchun, 130117 Jilin China; 3 Innovation Practice Center, Changchun University of Chinese Medicine, Changchun, 130117 Jilin China; 4 The Affiliated Hospital of Changchun University of Chinese Medicine, Changchun, 130117 Jilin China

**Keywords:** Chinese medicinal formula, Guzhi Zengsheng Zhitongwan, Serum proteomics, ITRAQ, Joint diseases, Molecular mechanism

## Abstract

**Background:**

Guzhi Zengsheng Zhitongwan (GZZSZTW) is an effective formula of traditional Chinese herbal medicine and has been widely applied in the treatment of joint diseases for many years. The aim of this study was to dissect the molecular targets and signaling pathways of Guzhi Zengsheng Zhitongwan based on the analysis of serum proteomics.

**Methods:**

The Chinese herbs of GZZSZTW were immersed in 5 l distilled water and boiled with reflux extraction method. The extract was filtered, concentrated and freeze-dried. The chemical profile of GZZSZTW extract was determined by high-performance lipid chromatography (HPLC). The 7-week old Sprague-Dawley (SD) rats in GZZSZTW groups were received oral administration at doses of 0.8, 1.05, and 1.3 g/kg per day and the rats in blank group were fed with drinking water. Serum samples were collected from the jugular veins. Primary chondrocyte viability was evaluated by CCK-8 assay. A full spectrum of the molecular targets and signaling pathways of GZZSZTW were investigated by isobaric tags for relative and absolute quantitation (iTRAQ) analysis and a systematic bioinformatics analysis accompanied with parallel reaction monitoring (PRM) and siRNA validation.

**Results:**

GZZSZTW regulated a series of functional proteins and signaling pathways responsible for cartilage development, growth and repair. Functional classification analysis indicated that these proteins were mainly involved in the process of cell surface dynamics. Pathway analysis mapped these proteins into several signalling pathways involved in chondrogenesis, chondrocyte proliferation and differentiation, and cartilage repair, including hippo signaling pathway, cGMP-PKG signaling pathway, cell cycle and calcium signaling pathway. Protein–protein interaction analysis and siRNA knockdown assay identified an interaction network consisting of TGFB1, RHO GTPases, ILK, FLNA, LYN, DHX15, PKM, RAB15, RAB1B and GIPC1.

**Conclusions:**

Our results suggest that the effects of GZZSZTW on treating joint diseases might be achieved through the TGFB1/RHO interaction network coupled with other proteins and signaling pathways responsible for cartilage development, growth and repair. Therefore, the present study has greatly expanded our knowledge and provided scientific support for the underlying therapeutic mechanisms of GZZSZTW on treating joint diseases. It also provided possible alternative strategies for the prevention and treatment for joint diseases by using traditional Chinese herbal formulas.

## Background

Guzhi Zengsheng Zhitongwan (GZZSZTW), which consists of *Spatholobus suberectus* Dunn, *Rehmannia glutinosa* (Gaertn.) DC., *Raphanus sativus* L. (Hook. f. & T. Anderson) (baked), *Epimedium brevicornu Maxim* (K. S. Hao), *Cynomorium coccineum* subsp. *Songaricum* (*Rupr.*) (J. Léonard), *Drynaria fortune* (Kunze ex Mett.) J.Sm. (baked) and *Cibotium barometz* (L.) (J.Sm), is an effective formula of traditional Chinese herbal medicine and has been widely applied in the treatment of joint diseases for many years. Our previous studies have shown that the effects of GZZSZTW may be partially achieved via controlling chondrocyte proliferation and differentiation by modulating genes and proteins involved in chondrocyte structure, dynamics, and metabolism [1, 2]. However, the precise molecular mechanism underlying its efficacy in treating joint diseases remains to be elucidated.

Cartilage, which is formed by chondrocytes, is an avascular, aneural, alymphatic connective tissue found in the body. Hyaline cartilage, which is predominantly found in the articulating surfaces of bones in synovial joints, is the most common form of cartilage in the human body [3]. Cartilage development begins with the condensation of undifferentiated mesenchymal cells, which is a prerequisite for subsequent chondrogenic differentiation [4]. SOX trio proteins including SOX5, SOX6 and SOX9 are master regulatory factors for chondrogenic differentiation and cartilage formation [5, 6]. Numerous growth factors and signaling pathways work in concert to regulate cartilage development, growth, maintenance and repair, such as TGFB, BMP7, IGF1 and FGF2 [7–10]. Among these molecules, the factors of TGFB family play a prominent role in the process of chondrogenesis, maintenance of cartilage homeostasis as well as cartilage repair [11]. TGFB is the main initiator of mesenchymal cell condensation during chondrogenesis, and then plays a key role in stimulating chondrocyte proliferation and extracellular matrix production while inhibiting chondrocyte hypertrophy and maturation [12, 13]. During these processes, TGFB stabilizes SOX9 protein, and SOX9 is sufficient and necessary for TGFB-mediated regulation of chondrogenesis [14–16]. Among the three identified TGFB isoforms in mammalian tissues including TGFB1, TGFB2 and TGFB3, TGFB1 is considered to be the predominant form of TGFB in articular cartilage [17]. Furthermore, Dysregulation of TGFB signaling and responses has been shown to be involved in osteoarthritis [18].Therefore, growth factors like TGFB have become the potential targets for cartilage repair and regeneration in the treatment of osteoarthritis [19, 20].

In recent years, proteomics has been widely used to detect functional proteins, drug targets and molecular mechanism of action in living systems under physiology and pathology conditions [21–23]. Furthermore, proteomics has made outstanding contributions to discover molecular targets and elucidate the underlying mechanisms of traditional Chinese medicine [24–26]. Serum contains complexity and dynamic functional proteins which fluctuate depending on the physiological and pathological conditions of living systems. Protein types and relative concentrations can be easily detected by performing serum proteomics analysis. Thus, serum was recognized as a highly believable sample for discoveries of drug targets and molecular mechanism of the action of drug in living systems under physiology and pathology conditions. Therefore, serum proteomics analysis plays a pivotal role in exploring the molecular mechanism of the action of drug [27–29].

In the present study, we performed quantitative proteomics analysis to investigate the effects of GZZSZTW on rat serum proteins using a state-of-the-art isobaric labeling method namely iTRAQ (isobaric tags for relative and absolute quantitation). Our findings indicated that the effects of GZZSZTW on treating joint diseases might achieved though the TGFB1/RHO interaction network coupled with other proteins and signaling pathways responsible for chondrogenesis, chondrocyte proliferation and differentiation and cartilage repair.

## Methods

### GZZSZTW preparation

The Chinese herbs of GZZSZTW, including *Spatholobus suberectus* Dunn (93 g), *Rehmannia glutinosa* (Gaertn.) DC. (139 g), *Raphanus sativus* L. (Hook. f. & T. Anderson) (baked) (46 g), *Epimedium brevicornu Maxim* (K. S. Hao) (93 g), *Cynomorium coccineum* subsp. *songaricum* (*Rupr.*) (J. Léonard) (93 g), *Drynaria fortunei* (Kunze ex Mett.) J.Sm. (baked) (93 g) and *Cibotium barometz* (L.) (J.Sm) (93 g) were provided by the Affiliated Hospital of Changchun University of Chinese Medicine (Changchun, China). The preparation of the GZZSZTW aqueous extract was carried out as previously described [1, 2]. Briefly, all of the herbal components were immersed in 5 L distilled water and boiled with reflux extraction method. The extract was filtered, concentrated, freeze-dried, and stored at − 80 °C until use.

### HPLC analysis of GZZSZTW Extract

The chemical profile of GZZSZTW extract was determined by high-performance lipid chromatography (HPLC). Standard chemicals (acteoside, epicatechin, icariin, rutin, naringin, protocatechuic acid and protocatechuic aldehyde) were purchased from National Institute for Food and Drug Control (Beijing, China). HPLC analysis of GZZSZTW extract was performed using a 2695 liquid chromatography system (Waters, USA) equipped with a reverse phase Symmetry C18 Column (Waters, USA). The mobile phase was a gradient elution system consisted of water containing 0.1% (v/v) formic acid (A) and acetonitrile (B) with a flow rate of 1 ml/min as following: 0–20 min, 95–85% A; 20–35 min, 85–75% A; 35–50 min, 75–55% A; 50–70 min, 55–10% A. The photodiode array (PDA) detector was set at 254 nm, and the on-line UV spectra were recorded in the range of 195–400 nm.

### Animals and treatment

All procedures were performed in accordance with the guidelines of the Institutional Animal Ethics Committee of Changchun University of Chinese Medicine (No. ccucm-2017-0015). Forty male Sprague-Dawley (SD) rats (7-week old, 200–250 g, SPF grade) were purchased from Changchun Yisi Laboratory Animal Technology Co, Ltd. (Certification number SCXK (Ji) 2016-0003, Changchun, China). Rats were housed in an air conditioned room at constant room temperature (25 ± 2 °C) and air humidity (50 ± 10%), with a 12-h light and dark cycle. Rats were randomly divided into blank group and GZZSZTW groups at different doses (10 rats per group) after being acclimatized to the facilities for 7 days. According to the calculation based on normalization to interspecies differences in body surface area [30], the dose selected for GZZSZTW in animal experiment should be 1.05 g/kg per day, which is the equivalent dosage used clinically for humans. Therefore, the rats in GZZSZTW groups were received oral administration at doses of 0.8, 1.05, and 1.3 g/kg per day and the rats in blank group were fed with drinking water.

### Serum collection and determination of effects on chondrocyte viability

All rats were anesthetized intraperitoneally by injecting chloral hydrate (400 mg/kg body weight) after 3 weeks of treatment. Serum samples were collected from the jugular veins as previously described [31]. All animals were euthanized by cervical dislocation at the completion of serum collection. Primary chondrocytes were isolated from the rib cages of new-born rats as previously described [32]. Briefly, cartilage from the rib cages was digested for 30 min with 0.25% trypsin (Sigma, USA) and 0.2% collagenase P (Sigma, USA), and then 60 min with 0.2% collagenase P (Sigma, USA). The cells were collected by centrifuging at 250×*g* for 5 min, and then re-suspended in DMEM/F12 complete culture medium (Thermo, USA) containing 5% fetal bovine serum (Thermo, USA), 100 U/ml ampicillin and 100 U/ml streptomycin (Sigma, USA).The effects of GZZSZTW-medicated serum at different doses on primary chondrocyte proliferation were assessed using the CCK-8 assay according to the manufacturer’s protocol. Briefly, primary chondrocytes were plated at a density of 5000 cells per well in a 96-well plate and cultured for 4 h at 37 °C in a humidified incubator with 5% CO_2_ (Thermo, USA). The cells were washed thoroughly with new DMEM/F12 medium after aspirating away the supernatant. The chondrocytes were treated with 10, 15 and 20% GZZSZTW-medicated serum diluted in culture medium, which were collected from rats at different doses (0.8, 1.05, and 1.3 g/kg) and subsequently cultured for 24 h. Following this, 20 µl of CCK-8 reagent was added to each well and incubated for 1 h. The absorbance was detected using an automatic Infinite 200 PRO microplate reader (Life Sciences, USA) at a wavelength of 450 nm. The relative proliferation rate was plotted using the percentage of viable cells (cell viability) at different concentrations under antler extracts treatment.

### Serum protein collection, reduction and alkylation

The serum proteins with high abundance were depleted for minimizing the interference by using a commercial ProteoMiner™ Protein Enrichment Kit (Bio-Rad, USA) following the manufacturer’s instructions. Serum proteins from the blank group and GZZSZTW group were pool together, separately. Protein samples were diluted in the lysis buffer containing 8 M urea and 2% CHAPS and reduced with 10 mM DTT (Sigma, USA) at 56 °C for 1 h. The samples were alkylated with 55 mM IAM (Sigma, USA) in the darkroom for 45 min. The protein mixture was precipitated by adding 5× volume of chilled acetone and incubated at − 20 °C for 2 h. After centrifugation for 15 min at 4 °C, the supernatant was discarded, and the pellet was suspended in 0.5 M TEAB (Sigma, USA) to dissolve the proteins and the protein mixture was sonicated in an ice-bath for 20 min. The supernatant protein was collected by centrifuging for 15 min at 4 °C. The proteins were quantified using the Bradford assay [33].

### Protein digestion, iTRAQ labeling and peptide fractionation

For each sample, 100 μg of total protein was digested with trypsin (Sigma, USA) with amass ratio of protein: trypsin = 20: 1 for 12 h at 37 °C. Following the enzyme digestion, the peptides were freeze-driedby vacuum centrifugation and reconstituted in 0.5 MTEAB (Sigma, USA). iTRAQ labeling was performed by using the iTRAQ Reagent 8-Plex One Assay Kit (AB Sciex, USA) following the manufacturer’s protocol. Peptides from different groups were labeled with different iTRAQ tags as follow: GZZSZTW group (tag 116), blank group (tag 113). The tagged samples were pooled together and dried by vacuum centrifugation. The iTRAQ-labeled peptide mixtures were reconstituted with 4 mL strong cation exchange (SCX) buffer A (25 mM NaH_2_PO_4_ in 25% acetonitrile (ACN), pH 2.7) and loaded onto a LC-20AB high-performance liquid chromatography platform (Shimadzu, Japan) equipped with a 4.6 × 250 mm Ultremex strong cation exchange column containing 5‐μm particles (Phenomenex, USA) [2].

### Nano-HPLC–MS/MS analysis

Each fraction was further subjected to a TripleTOF 5600 mass spectrometer system (AB SCIEX, USA) equipped with a nanoACQuity UPLC system (Waters, USA) according to previously described [2]. Briefly, the peptide sample from each SCX fraction was loaded onto a nanoACQuity UPLC BEH130 column (Waters, USA) packed with Symmetry C18 resin (Waters, USA). A Triple TOF 5600 platform was used for peptide identification. The ion spray voltage was set at 2.5 kV, the curtain gas was set at 30 psi, the nebulizer gas was set at 15 psi, and the interface heater temperature was set at 150 °C, respectively. For TOF–MS scans, the resolving power (RP) was greater than or equal to 30,000 FWHM. 250 ms was required for survey scans for the information dependent acquisition (IDA) analysis. 30 products were collected if the ion scans were more than 120 counts per second and with a 2+ to 5+ charge state. The Q2 transmission window was set at 100 Da for 100%. A sweeping collision energy setting of 35 ± 5 eV coupled with iTRAQ adjusted rolling collision energy was applied to all precursor ions for collision-induced dissociation. The parent ion dynamic exclusion was set to half of the peak time, and then the precursor was refreshed off the exclusion list.

### Data processing

The Mascot (Matrix Sciences, UK) and Proteome Discoverer software (Thermo, USA) were used for protein identification as previously described [2]. The collected raw data were converted into MGF files using the Proteome Discoverer software. Protein identifications were conducted by searching against the NCBI nr (https://www.ncbi.nlm.nih.gov/refseq/) and UniProt protein databases (http://www.uniprot.org/) using the Mascot program. The multiple search results were combined by the IPeak software and quantified by the IQuant software. Proteins with a statistically significant fold change ≥ 1.5 or ≤ 0.67 (*p* value ≤ 0.05) were considered to be differentially expressed proteins. Function and pathway enrichment analyses were further performed by searching the differentially expressed proteins against the Gene Ontology (GO) (http://www.geneontology.org/) and Kyoto Encyclopedia of Genes and Genomes (KEGG) (http://www.genome.jp/kegg/pathway.html) databases. Only a *p* value < 0.05 was considered as significant enrichment. Search Tool for the Retrieval of Interacting Genes/Proteins (STRING) (https://string-db.org/) was then used for protein–protein interaction analysis.

### Parallel reaction monitoring validation

The protein expression levels obtained by the iTRAQ analysis were further validated by the parallel reaction monitoring (PRM) assays as previously described [2]. Briefly, the spectral library was generated by the SpectroDive software (Biognosys AG, Switzerland), and the candidate proteins were obtained from the Data Dependent Acquisition (DDA) analysis mode that were generated by a Q-EXACTIVE mass spectrometer system (Thermo, USA). Data analysis was carried out according to the default analysis mode with minor modifications. Student’s t-test was used to compare the significances of differences according to the fold changes of protein expression levels, with a *p* value equal or smaller than 0.05.

### siRNA knockdown assay

Primary chondrocytes were isolated and seeded into 96-well or 24-well plates (4000 or 50,000 cells per well) and cultured overnight. Silencer^®^ Select pre-designed TGFB1 (s75041, Thermo, USA) and negative control siRNAs (4390843, Thermo, USA) were transiently transfected with a final concentration of 10 nM each siRNA into primary chondrocytes using Lipofectamine^®^ RNAiMAX reagent (Thermo, USA) and Opti-MEM^®^ Medium according to the manufacturer’s protocol. After 24 h of transfection, cells were replenished with complete medium in the presence or absence of GZZSZTW-medicated serum for an additional 24 h, and processed for qRT-PCR and CCK-8 analyses.

## Results

### Extract yield and chemical quality control of GZZSZTW aqueous extract

**Fig. 1:**
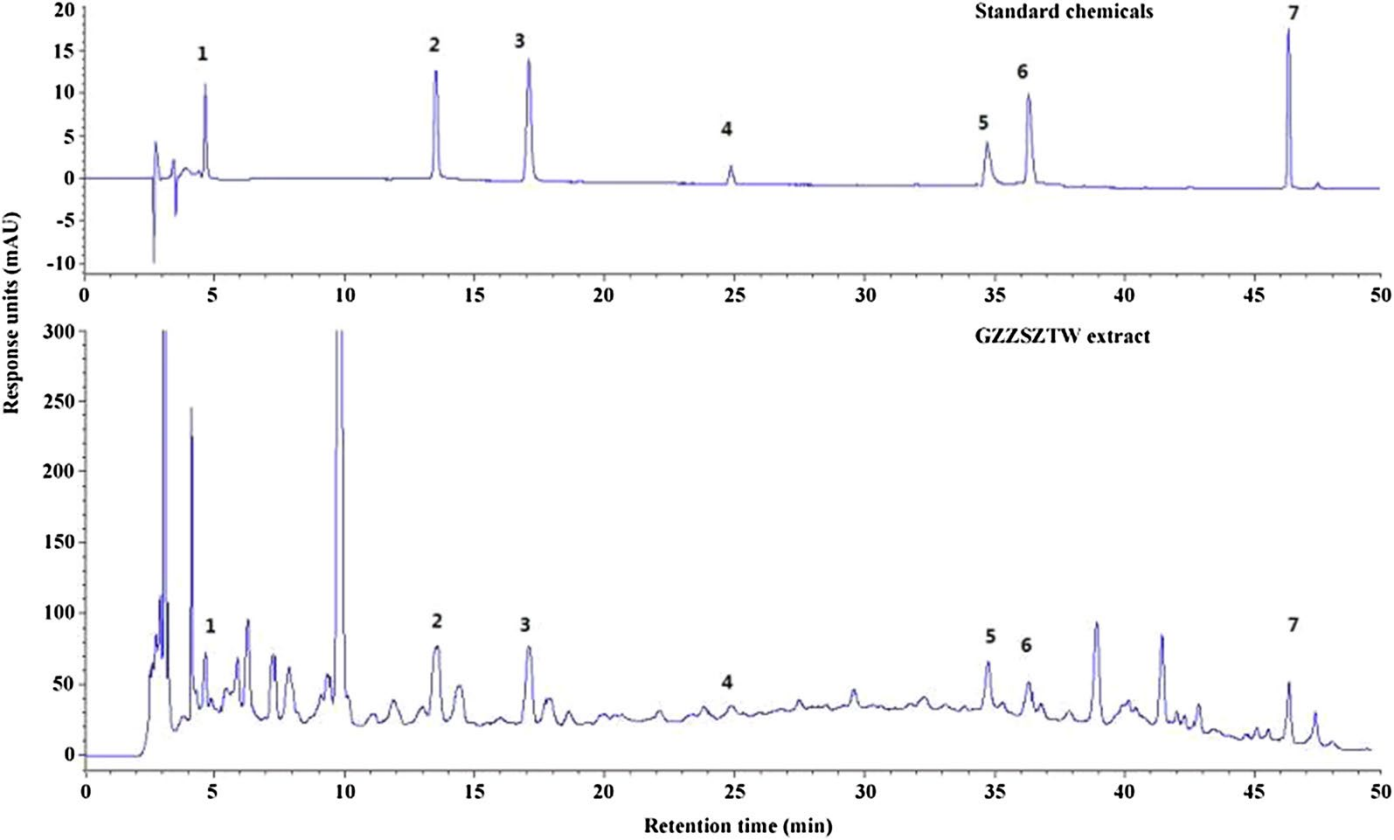
High performance liquid chromatography (HPLC) of standard chemicals (upper panel) and GZZSZTW extract (lower panel). Seven major components (1 to 7) were identified, 1: naringin; 2: protocatechuic acid; 3: protocatechuic aldehyde; 4: epicatechin; 5: acteoside; 6: rutin; 7: icariin. The ordinate represents the signal response units (mAU), while the abscissa represents the retention time (min)

In this study, the extract yield of GZZSZTW aqueous extract was 150.708 ± 0.86 g (23.18 ± 0.13%), and the chemical quality control was shown in Fig. 1 and Table 1. The chemical compounds except the icariin within *Epimedium brevicornu Maxim* and the rutin within *Raphanus sativus* L. showed consistent levels of contents with the previously reported standards, such as acteoside within *Rehmannia glutinosa*, epicatechin within *Spatholobus suberectus*, naringin within *Drynaria fortunei*, protocatechuic acid within *Cynomorium coccineum subsp. Songaricum* and protocatechuic aldehyde within *Cibotium barometz*.

**Table 1:** Main components and content assay of GZZSZTW aqueous extract

Herb name	Content assay	Standard (%)	GZZSZTW (%)
*Rehmannia glutinosa* (Gaertn.) DC.	Acteoside	≥ 0.020 [34]	0.209
*Spatholobus suberectus* Dunn	Epicatechin	≥ 0.017 [35]	0.121
*Epimedium brevicornu Maxim* (K. S. Hao)	Icariin	≥ 0.187 [36]	0.075
*Raphanus sativus* L. (Hook. f. & T. Anderson) (baked)	Rutin	≥ 0.600 [37]	0.112
*Drynaria fortunei* (Kunze ex Mett.) J.Sm. (baked)	Naringin	≥ 0.09 [38]	0.365
*Cynomorium coccineum subsp. songaricum* (*Rupr.*) (J. Léonard)	Protocatechuic acid	≥ 0.100 [39]	0.411
*Cibotium barometz* (L.) (J.Sm)	Protocatechuic aldehyde	≥ 0.033 [40]	0.410

### GZZSZTW-medicated serum stimulates chondrocyte proliferation in a dose-dependent manner

**Fig. 2:**
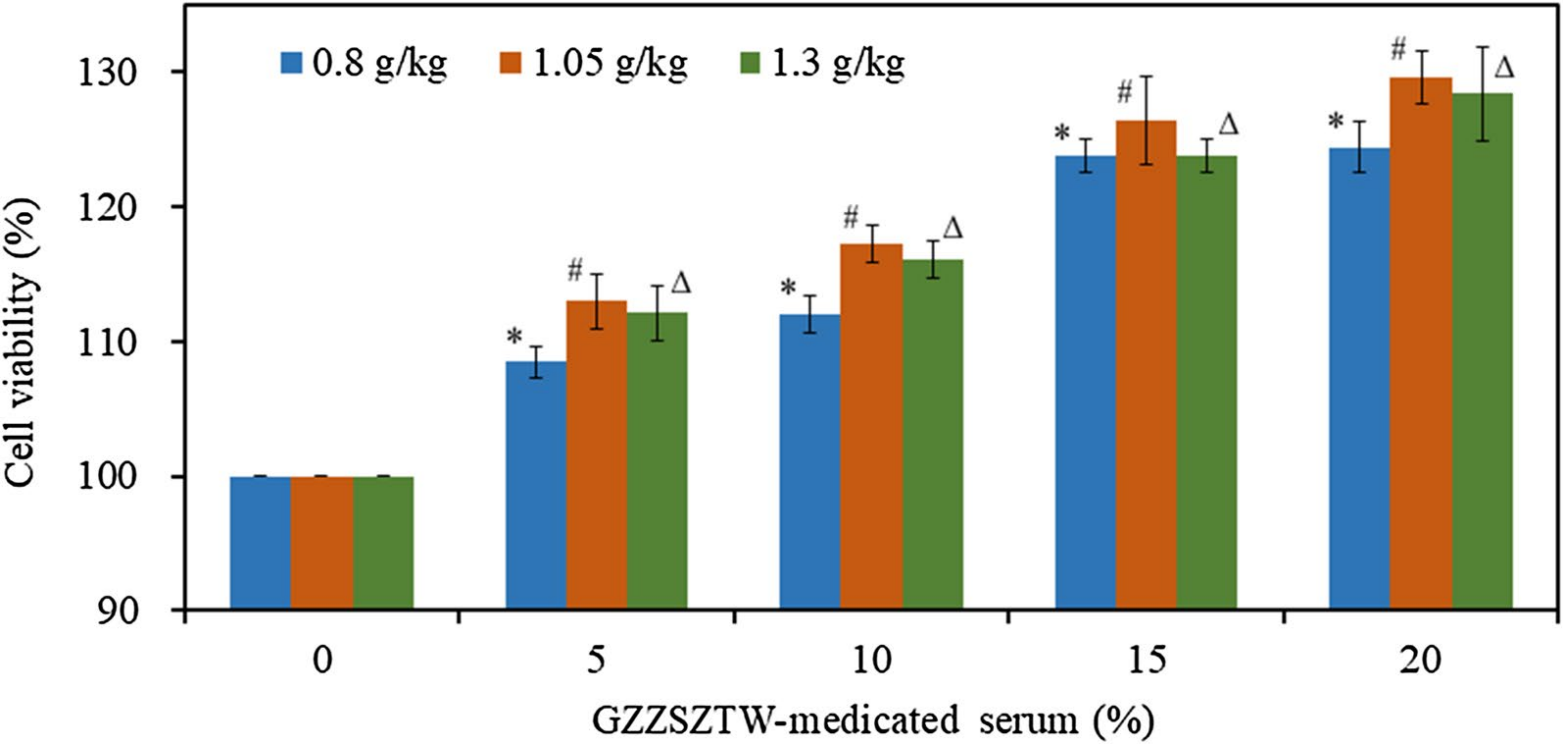
Effects of GZZSZTW-medicated serum on the proliferation of primary chondrocytes. CCK-8 assay was used to detect the chondrocyte proliferation following treatment with GZZSZTW-medicated serum at increasing concentrations (0%, 5%, 10% and 20%) for 24 h. The ordinate represents the cell viability (%), while the abscissa represents the concentration of GZZSZTW-medicated serum (%) in the culture of primary chondrocytes. Cell viability of the treatment of GZZSZTW-medicated serum was normalized and calculated relative to that of the blank group (0%). Data are presented as the mean of three independent experiments for technical triplicates with standard deviation.*, # and Δ represent *p *< 0.001 in a t-test for the difference in cell viability under the treatment of GZZSZTW-medicated serum collected at different doses including 0.8, 1.05 and 1.3 g/kg

The effect of GZZSZTW-medicated serum on primary chondrocyte proliferation was measured by the CCK-8 assay. As shown in Fig. 2, the chondrocyte viability was significantly increased under the treatment of GZZSZTW-medicated serum in a dose-dependent manner compared with the blank group (0%). Since treatment with GZZSZTW-medicated serum at the dose of 1.05 g/kg showed much higher chondrocyte viability compared to the other two doses, the serum collected at a dose of 1.05 g/kg GZZSZTW was selected for further use in the following experiments.

### Identification of differentially expressed serum proteins under GZZSZTW treatment

According to the analyses of iTRAQ-based quantitative proteomics between blank group and GZZSZTW treated group, a total of 171 differentially expressed proteins were identified using stringent criteria (fold change ≥ 1.5 or ≤ 0.67, *p* value ≤ 0.05). Among these differentially expressed proteins, the expression levels of 48 proteins were significantly increased under GZZSZTW treatment with a fold change more than 2, as shown in Table 2, such as DHX15, GREB1, AFF3, CROCC and GAPDH, etc. The expression levels of 97 proteins were significantly increased under GZZSZTW treatment with a fold change ranged from 1.5 to 2, as shown in Table 3, such as FAM50B, PIP4K2A, EEF1G, TAOK3 and CDC42, etc. The expression levels of 26 proteins were significantly decreased under GZZSZTW treatment with a fold change ≤ 0.67, as shown in Table 4, such as KNG1, CCDC85C, CES2A, ZFHX2 and PNKD, etc.

**Table 2:** Up-regulated proteins with a fold change > 2.0 under GZZSZTW treatment (GZZSZTW vs. Blank)

Protein name	Fold change	*p* value
Pre-mRNA-splicing factor ATP-dependent RNA helicase DHX15 (DHX15)	7.11	0.01
Protein GREB1 (GREB1)	4.68	0.02
AF4/FMR2 family member 3 (AFF3)	3.85	0.03
Rootletin (CROCC)	3.55	0.04
Glyceraldehyde-3-phosphate dehydrogenase (GAPDH)	3.31	0
Fibrinogen beta chain (FGB)	3.19	0
Chromodomain-helicase-DNA-binding protein 1-like (CHD1L)	3.11	0
Sodium bicarbonate transporter-like protein 11 (SLC4A11)	3.08	0
Fibrinogen gamma chain (FGG)	2.82	0
Calmodulin-regulated spectrin-associated protein 1 (CAMSAP1)	2.71	0
Four and a half LIM domains protein 1 (FHL1)	2.68	0
MOB kinase activator 1A (MOB1A)	2.65	0
Tubulin alpha-4A chain (TUBA4A)	2.58	0
Myelin regulatory factor (MYRF)	2.57	0
Von Ebner gland protein 1 (VEGP1)	2.56	0.01
Pro-interleukin-16 (IL16)	2.47	0
Elongation factor 1-beta (EEF1B)	2.45	0.01
Transgelin-2 (TAGLN2)	2.41	0
Destrin (DSTN)	2.40	0
Coronin-1A (CORO1A)	2.40	0
WD repeat-containing protein 1 (WDR1)	2.39	0
Flavin reductase (NADPH) (BLVRB)	2.35	0.01
Inorganic pyrophosphatase (PPA1)	2.35	0.01
Tubulin beta chain (TUBB)	2.35	0
Adenylyl cyclase-associated protein 1 (CAP1)	2.34	0
Disks large-associated protein 4 (DLGAP4)	2.29	0.01
EH domain-containing protein 3 (EHD3)	2.26	0
T-complex protein 1 subunit zeta (CCT6A)	2.25	0
Carboxypeptidase B2 (CPB2)	2.24	0
Beta-parvin (PARVB)	2.23	0
Profilin-1 (PFN1)	2.21	0
Pleckstrin (PLEK)	2.18	0
LIM and senescent cell antigen-like-containing domain protein 1 (LIMS1)	2.17	0
Inverted formin-2 (INF2)	2.16	0.01
Dynein light chain 1, cytoplasmic (DYNLL1)	2.15	0
Protein S100-A4 (S100A4)	2.15	0.02
Cysteine and glycine-rich protein 1 (CSRP1)	2.14	0.04
Filamin-A (FLNA)	2.10	0
Peptidyl-prolyl *cis*-*trans* isomerase A (PPIA)	2.10	0
Transitional endoplasmic reticulum ATPase (VCP)	2.10	0
Myosin light chain kinase family member 4 (MYLK4)	2.09	0.02
Calmodulin-2 B (CALM2B)	2.07	0
Alpha-actinin-1 (ACTN1)	2.07	0
Microtubule-associated protein RP/EB family member 2 (MAPRE2)	2.07	0
GMP reductase 1 (GMPR)	2.04	0.01
Aspartate-tRNA ligase, cytoplasmic (DARS)	2.03	0.03
Cofilin-1 (CFL1)	2.02	0
Hsc70-interacting protein (ST13)	2.01	0

**Table 3:** Up-regulated proteins with a fold change ≥ 1.5 and ≤ 2.0 under GZZSZTW treatment (GZZSZTW vs. Blank)

Protein name	Fold change	*p* value
Protein FAM50B (FAM50B)	2.00	0
Phosphatidylinositol 5-phosphate 4-kinase type-2 alpha (PIP4K2A)	2.00	0
Elongation factor 1-gamma (EEF1G)	1.98	0
Serine/threonine-protein kinase TAO3 (TAOK3)	1.98	0.03
Cell division control protein 42 homolog (CDC42)	1.98	0
Ras-related protein Rab-27B (RAB27B)	1.97	0
Protein S100-A9 (S100A9)	1.97	0.01
cGMP-specific 3′,5′-cyclic phosphodiesterase (PDE5A)	1.96	0.01
Pyruvate kinase PKM (PKM)	1.96	0
Pyrethroid hydrolase Ces2e (CES2E)	1.95	0.01
Coronin-1B (CORO1B)	1.95	0
T-complex protein 1 subunit epsilon (CCT5)	1.93	0
Caveolae-associated protein 2 (CAVIN2)	1.93	0
Septin-9 (SEPT9)	1.93	0.04
14-3-3 protein eta (YWHAH)	1.92	0
Regulator of G-protein signaling 18 (RGS18)	1.92	0.04
Heat shock cognate 71 kDa protein (HSPA8)	1.92	0
Talin-1 (TLN1)	1.92	0
Ras-related protein Rap-1b (RAP1B)	1.92	0
Src kinase-associated phosphoprotein 2 (SKAP2)	1.91	0
Glucose-6-phosphate 1-dehydrogenase (G6PDX)	1.91	0
UMP-CMP kinase (CMPK1)	1.89	0
Guanylate cyclase soluble subunit beta-1 (GUCY1B3)	1.88	0.01
Glutathione peroxidase 1 (GPX1)	1.88	0
Serine/threonine-protein kinase 26 (STK26)	1.88	0
Chloride intracellular channel protein 1 (CLIC1)	1.88	0
Vitamin D-binding protein (GC)	1.88	0
14-3-3 protein zeta/delta (YWHAZ)	1.88	0
Tubulin beta-7 chain (TUBB7)	1.87	0
Tubulin alpha-1C chain (TUBA1C)	1.86	0
Tubulin beta-2A chain (TUBB2A)	1.86	0
Galectin-1 (LGALS1)	1.85	0
Myosin regulatory light chain RLC-A (RLCA)	1.85	0
Eukaryotic translation initiation factor 5A-2 (EIF5A2)	1.84	0
Dynamin-2 (DNM2)	1.84	0
T-complex protein 1 subunit beta (CCT2)	1.83	0
Tropomyosin alpha-4 chain (TPM4)	1.82	0
Alpha-soluble NSF attachment protein (NAPA)	1.81	0
Elongation factor 1-delta (EEF1D)	1.80	0
Peroxiredoxin-1 (PRDX1)	1.80	0
Vinculin (VCL)	1.79	0
Tropomyosin beta chain (TPM2)	1.79	0
Arachidonate 12-lipoxygenase, 12S-type (ALOX12)	1.78	0
Serine protease inhibitor A3 M (SERPINA3 M)	1.77	0
Creatine kinase B-type (CKB)	1.76	0
Alpha-centractin (ACTR1A)	1.76	0
Renin receptor (ATP6AP2)	1.75	0
Nuclear receptor-binding protein (NRBP1)	1.75	0.01
Tyrosine-protein kinase Lyn (LYN)	1.73	0.03
Cytosol aminopeptidase (LAP3)	1.72	0.01
UV excision repair protein RAD23 homolog A (RAD23A)	1.72	0.01
Tropomyosin alpha-1 chain (TPM1)	1.71	0.01
Tubulin beta-1 chain (TUBB1)	1.70	0
Ras-related protein Rab-11B (RAB11B)	1.70	0
T-complex protein 1 subunit eta (CCT7)	1.69	0.01
Integrin-linked protein kinase (ILK)	1.69	0
Eukaryotic peptide chain release factor subunit 1 (ETF1)	1.69	0.02
Ras-related protein Rab-15 (RAB15)	1.68	0
T-complex protein 1 subunit gamma (CCT3)	1.68	0
cGMP-specific 3′,5′-cyclic phosphodiesterase (PDE5A)	1.67	0
Creatine kinase M-type (CKM)	1.67	0
Ras-related protein Rab-35 (RAB35)	1.67	0
Xaa-Pro aminopeptidase 1 (XPNPEP1)	1.66	0
GTP-binding nuclear protein Ran (RAN)	1.65	0
Moesin (MSN)	1.65	0
Class I histocompatibility antigen, Non-RT1.A alpha-1 chain (RT1AW2)	1.63	0
Glycogen phosphorylase, liver form (PYGL)	1.63	0
Transforming protein RhoA (RHOA)	1.63	0
Ras suppressor protein 1 (RSU1)	1.63	0.01
Ras GTPase-activating protein 3 (RASA3)	1.62	0
Myosin light polypeptide 6 (MYL6)	1.62	0
Actin, cytoplasmic 2 (ACTG1)	1.61	0
Lipopolysaccharide-binding protein (LBP)	1.60	0
l-lactate dehydrogenase A chain (LDHA)	1.60	0
Protein diaphanous homolog 1 (DIAPH1)	1.60	0.01
T-complex protein 1 subunit delta (CCT4)	1.60	0
cAMP-dependent protein kinase type II-beta regulatory subunit (PRKAR2B)	1.60	0
Ubiquitin carboxyl-terminal hydrolase 5 (USP5)	1.59	0
Ras-related protein Rab-1B (RAB1B)	1.57	0.02
Cytosolic non-specific dipeptidase (CNDP2)	1.57	0
Protein S100-A8 (S100A8)	1.56	0.01
PDZ domain-containing protein GIPC1 (GIPC1)	1.56	0.02
Fermitin family homolog 3 (FERMT3)	1.56	0
Tyrosine-protein phosphatase non-receptor type 11 (PTPN11)	1.56	0
Ras-related C3 botulinum toxin substrate 1 (RAC1)	1.55	0
Myosin-9 (MYH9)	1.55	0
Tropomyosin alpha-3 chain (TPM3)	1.55	0
AP-1 complex subunit beta-1 (AP1B1)	1.54	0
T-complex protein 1 subunit alpha (TCP1)	1.54	0
RAS guanyl-releasing protein 2 (RASGRP2)	1.53	0
Myosin light chain kinase, smooth muscle (MYLK)	1.53	0
Keratin, type II cytoskeletal 1 (KRT1)	1.52	0
Myosin regulatory light polypeptide 9 (MYL9)	1.52	0
Inhibin beta C chain (INHBC)	1.51	0
Ubiquitin-like modifier-activating enzyme 1 (UBA1)	1.51	0
14-3-3 protein beta/alpha (YWHAB)	1.50	0
Transforming growth factor beta-1 (TGFB1)	1.50	0

**Table 4:** Down-regulated proteins with a fold change ≤ 0.67 under GZZSZTW treatment (GZZSZTW vs. Blank)

Protein name	Fold change	*p* value
Kininogen-1 (KNG1)	0.22	0.02
Coiled-coil domain-containing protein 85C (CCDC85C)	0.22	0.02
Pyrethroid hydrolase Ces2a (CES2A)	0.27	0
Zinc finger homeobox protein 2 (ZFHX2)	0.28	0
Probable hydrolase PNKD (PNKD)	0.29	0.04
Apolipoprotein F (APOF)	0.29	0
Alpha-1B-glycoprotein (A1BG)	0.42	0
Serine protease hepsin (HPN)	0.43	0
Alcohol dehydrogenase 1 (ADH1)	0.44	0
Serine protease inhibitor A3N (SERPINA3N)	0.48	0
NF-kappa-B inhibitor zeta (NFKBIZ)	0.48	0
Thyrotropin-releasing hormone-degrading ectoenzyme (TRHDE)	0.48	0.02
Beta-2-glycoprotein 1 (APOH)	0.52	0
Glucosidase 2 subunit beta (PRKCSH)	0.52	0
Alpha-1-antiproteinase (SERPINA1)	0.55	0
Proprotein convertase subtilisin/kexin type 9 (PCSK9)	0.56	0
CD5 antigen-like (CD5L)	0.58	0
Apolipoprotein B-100 (APOB)	0.58	0
Angiopoietin-related protein 3 (ANGPTL3)	0.60	0
Ficolin-2 (FCN2)	0.60	0
T-kininogen 1 (MAP1)	0.61	0
Osteomodulin (OMD)	0.65	0
Keratinocyte differentiation-associated protein (KRTDAP)	0.66	0
Hyaluronan-binding protein 2 (HABP2)	0.66	0
Translationally-controlled tumor protein (TPT1)	0.66	0.02
Napsin-A (NAPSA)	0.66	0

### Functional classification of differentially expressed proteins

**Fig. 3:**
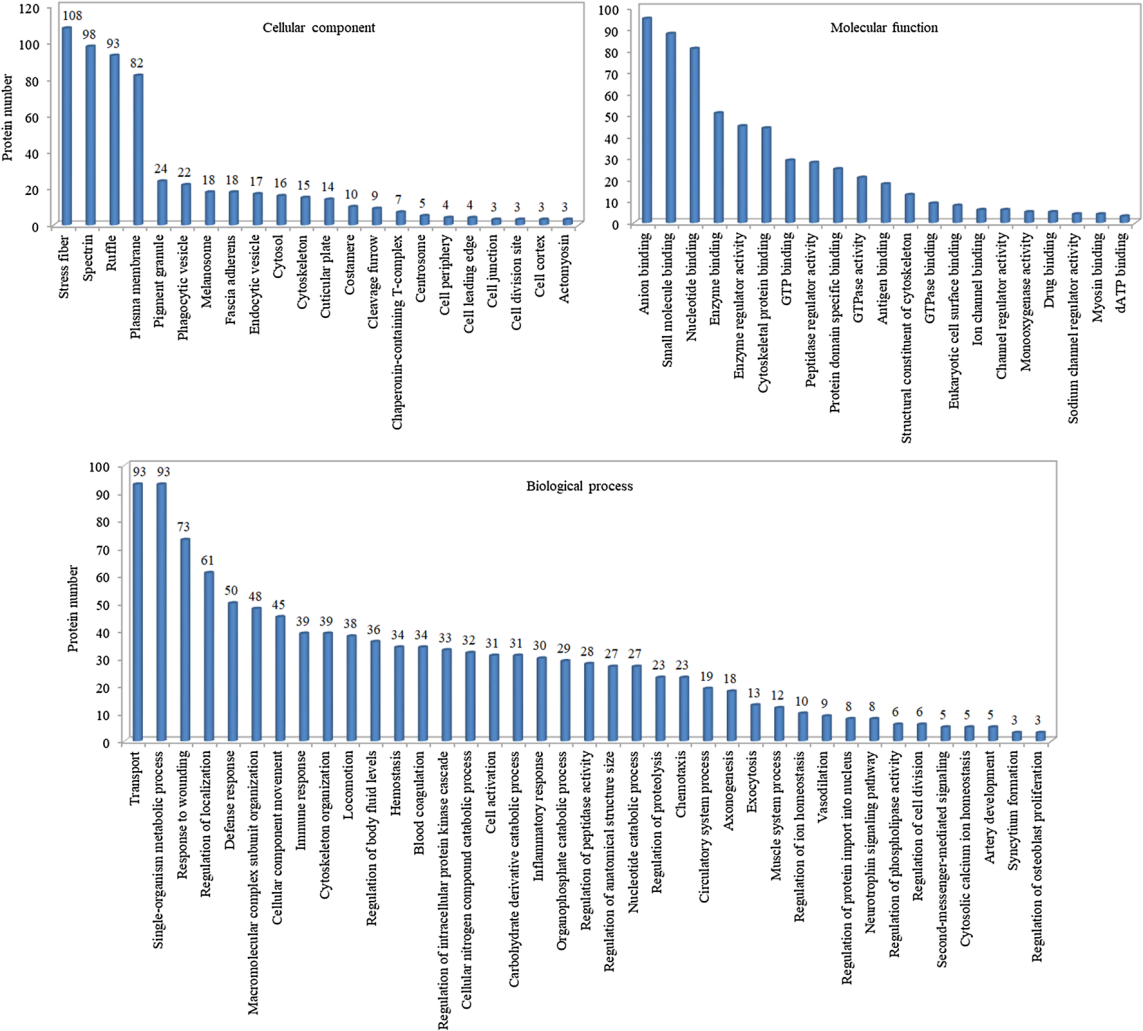
Histogram of GO classification of differentially expressed proteins. The results were grouped into three main categories: cellular component, molecular function and biological process. The ordinate represents the number of differentially expressed proteins corresponding to the GO term, while the abscissa represents the name of the GO term

To gain insight into the biological functions of the serum proteins under GZZSZTW treatment, the differentially expressed proteins were categorized according to the following GO classes: cellular component, molecular function and biological process, as shown in Fig. 3. Cellular component classification showed that most of the differentially expressed proteins were located in the regions of stress fiber, spectrin, ruffle and plasma membrane. Molecular function classification showed that the dominant functions of these proteins were anion binding, small molecule binding and nucleotide binding. Biological process classification showed that these proteins mainly participated in the processes of transport, single-organism metabolic process, response to wounding and regulation of localization.

### KEGG pathway analysis of differentially expressed proteins

**Fig. 4:**
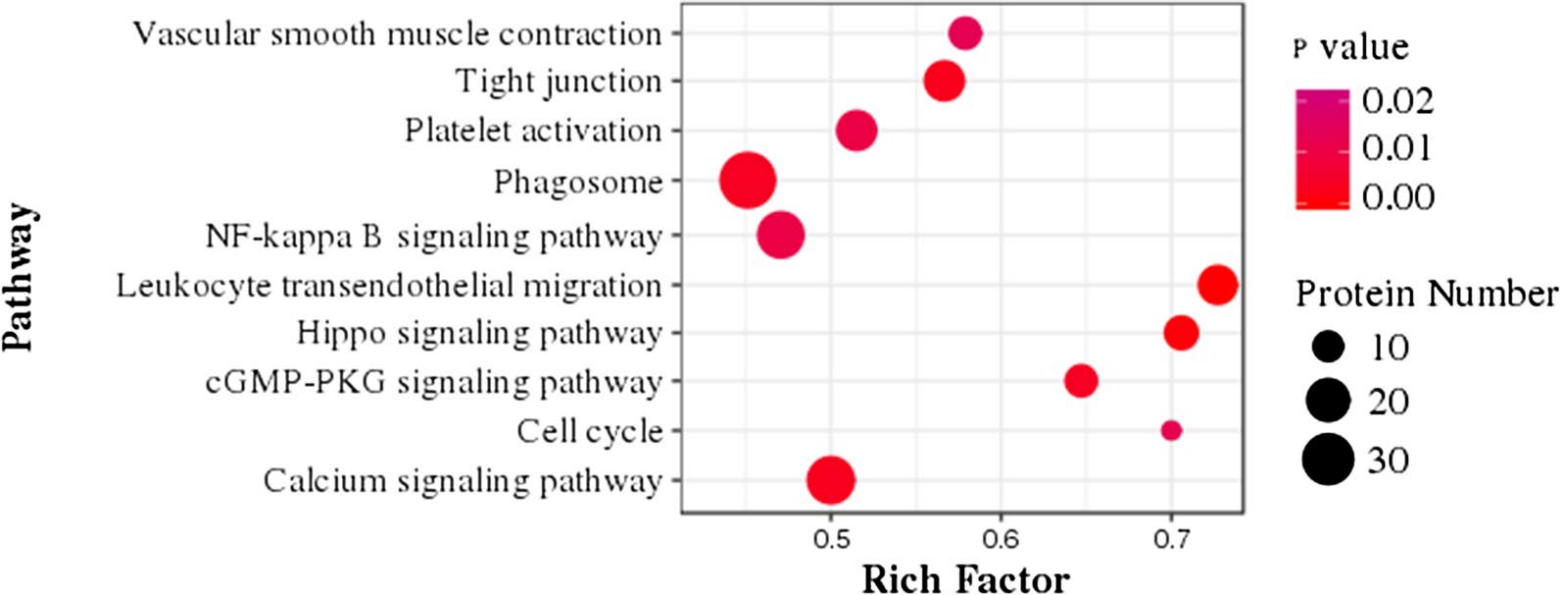
Scatter plot of KEGG pathways for differentially expressed proteins. The ordinate represents the enriched pathway, and the abscissa represents the rich factor, which means the number of differentially expressed proteins participating in a KEGG pathway as a proportion of proteins involved in the pathway in all identified proteins. The dot size indicates the number of differentially expressed proteins, and the dot color indicates the *p* value

To explore the possible physiological processes and pathways of the serum proteins under GZZSZTW treatment, we searched these proteins against the KEGG database. As shown in Fig. 4, the differentially expressed proteins mainly participated in the pathways including vascular smooth muscle contraction, tight junction, platelet activation, phagosome, NF-kappa B signaling pathway, leukocyte transendothelial migration, hippo signaling pathway, cGMP-PKG signaling pathway, cell cycle and calcium signaling pathway.

### Protein–protein interaction analysis of differentially expressed proteins

**Fig. 5:**
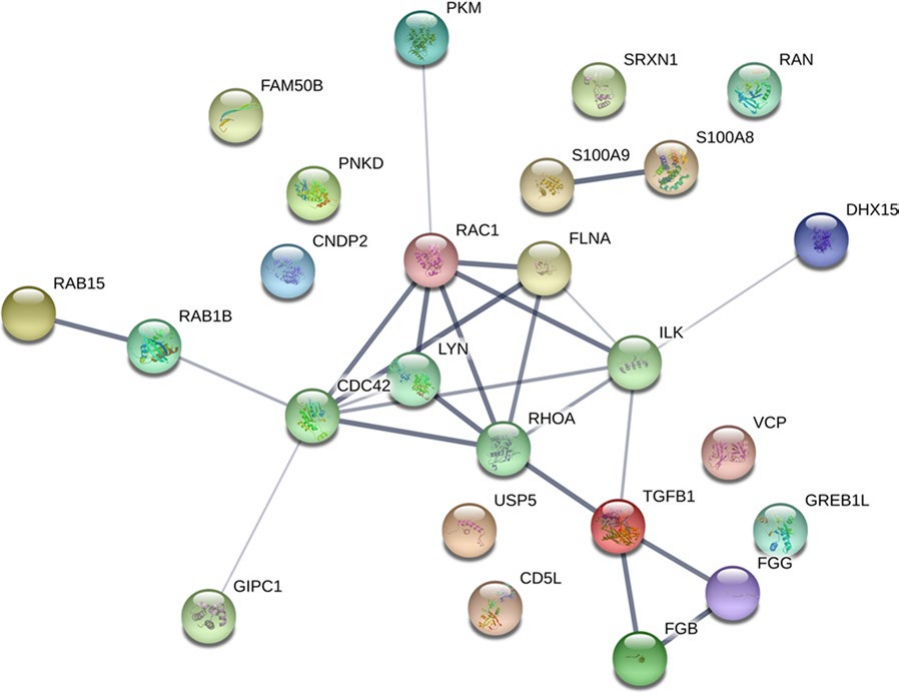
The interaction network of differentially expressed proteins. In this network, proteins are represented as nodes with different colours, and the lines connecting the nodes represent functional associations between proteins. Line thickness indicates the strength of confidence in the reported association

To further facilitate a better understanding of the molecular mechanism of the crosstalk among the differentially expressed proteins, both the up-regulated and down-regulated proteins were submitted to the STRING website to assess the protein–protein interaction network, as shown in Fig. 5. Among the up-regulated proteins, 23 proteins were mapped in the network, and 14 proteins were interconnected. In addition, TGFB1, RHOA, ILK, FLNA, RAC1, LYN and CDC42 were located in the center of the network and served as a hub to interact with other proteins. However, of the 26 identified down-regulated proteins, only 2 proteins (PNKD and CD5L) were mapped with no interaction with the network.

### PRM validation

The PRM analysis was carried out to validate the expression levels of iTRAQ identified differentially expressed proteins. 14 differentially expressed proteins within the identified protein–protein interaction network, including TGFB1, RHOA, ILK, FLNA, RAC1, LYN, CDC42, FGG, FGB, DHX15, PKM, RAB15, RAB1B and GIPC1, were chosen for the PRM analysis. The fold changes of differentially expressed proteins were consistent with those of the iTRAQ analysis, as shown in Table 5.

**Table 5:** Expression validation of differentially expressed proteins by PRM analysis (GZZSZTW vs. Blank)

Protein name	iTRAQ		PRM	
	Fold change	*p* value	Fold change	*p* value
TGFB1	1.50	0	2.14	0.01
RHOA	1.63	0	1.45	0
ILK	1.69	0	1.13	0.02
FLNA	2.10	0	3.41	0
RAC1	1.55	0	1.92	0.04
LYN	1.73	0.03	2.42	0
CDC42	1.98	0	1.79	0
FGG	2.82	0	1.84	0.03
FGB	3.19	0	2.34	0.01
DHX15	7.11	0.01	5.02	0.01
PKM	1.96	0	1.30	0.04
RAB15	1.68	0	1.70	0.03
RAB1B	1.57	0.02	1.86	0.02
GIPC1	1.56	0.02	1.83	0

#### Validation of protein–protein interaction network

**Fig. 6:**
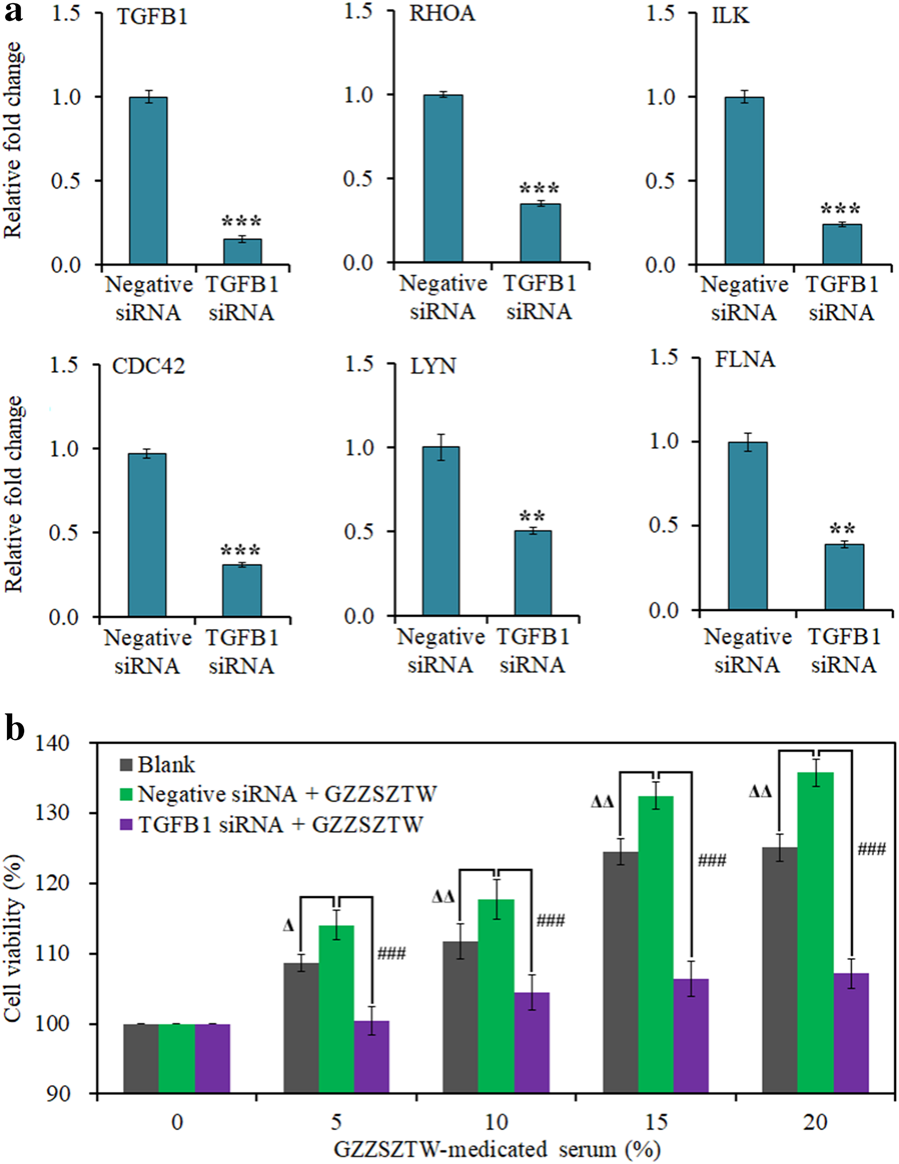
Validation of protein–protein interaction network by siRNA knockdown and CCK-8 assays. **a** Gene expression levels of TGFB1, RHOA, ILK, CDC42, LYN and FLNA validated by qRT-PCR under TGFB1 silencing in primary chondrocytes. Relative mRNA levels were normalized to the GAPDH level and calculated using the 2^−ΔΔCT^ method. The relative fold-changes of each gene in the TGFB1 siRNA group were normalized and compared to the Negative siRNA group, separately. Data are presented as the mean with standard deviation for technical triplicates in an experiment. * represents p < 0.01, and *** represents p < 0.001 in a t-test for the difference in gene expression level. **b** Effects of GZZSZTW-medicated serum on the proliferation of primary chondrocytes under TGFB1 silencing. CCK-8 assay was used to detect the chondrocyte proliferation following treatment with GZZSZTW-medicated serum at increasing concentrations (0%, 5%, 10% and 20%) for 24 h after siRNA knockdown. The ordinate represents the cell viability (%), while the abscissa represents the concentration of GZZSZTW-medicated serum (%) in the culture of primary chondrocytes. Cell viability was normalized and calculated relative to that of the blank group (0%). Data are presented as the mean of three independent experiments for technical triplicates with standard deviation. Δ and ΔΔ represent p < 0.05 and p < 0.01, and ### represents p < 0.001 in a t-test for the difference in cell viability (%)

Furthermore, we found that silencing of TGFB1 by siRNA significantly decreased the expression levels of RHOA, ILK, CDC42, LYN and FLNA. In addition, TGFB1 silencing significantly antagonized the effect of GZZSZTW-medicated serum on chondrocyte proliferation, as shown in Fig. 6.

## Discussion

GZZSZTW is a traditional Chinese medicinal formula created by the national medical master professor Bailing Liu. It has been widely used for treating joint diseases for many decades, such as osteoarthritis. In our previous studies, we have showed that the effects of GZZSZTW on treating joint diseases might be achieved through regulating chondrocyte proliferation and differentiation in a way of controlling functional genes and proteins involved in chondrocyte homeostasis [1, 2]. In the present study, we investigated the underlying mechanism of GZZSZTW on regulating rat serum proteome using state-of-the-art iTRAQ technology. We totally identified 171 differentially expressed proteins by performing the iTRAQ experiment. Among these identified proteins, a majority of these proteins (145, ~ 85%) were significantly increased under the treatment of GZZSZTW. There were 48 proteins with a fold change more than 2, and 97 proteins with a fold change ranged from 1.5 to 2.

By GO classification analysis, these identified proteins were mainly located in the regions of stress fiber, spectrin, ruffle and plasma membrane, and were predominantly involved in the functions of anion binding, small molecule binding and nucleotide binding. Furthermore, these proteins primarily participated in the biological processes of transport, single-organism metabolic process, response to wounding and regulation of localization. These results suggest that GZZSZTW treatment affect cell surface dynamics by regulating serum proteins responsible for molecular connections between the plasma membrane and the cytoskeleton. KEGG pathway analysis indicated that these proteins mainly participated in the pathways including vascular smooth muscle contraction, tight junction, platelet activation, phagosome, NF-kappa B signaling pathway, leukocyte transendothelial migration, hippo signaling pathway, cGMP-PKG signaling pathway, cell cycle and calcium signaling pathway. Vascular smooth muscle contraction, tight junction, platelet activation, phagosome and leukocyte transendothelial migration are pathways involved in vascular homeostasis, angiogenesis, inflammation and immune response [41–45]. NF-kappa B signaling pathway plays a key role in regulating the processes of inflammation and damage to articular cartilage and serve as a potential therapeutic target in treating osteoarthritis [46, 47]. Hippo signaling pathway controls organ size and tissue regeneration in many organs, and particularly regulates skeletal development and postnatal growth by controlling chondrocyte proliferation and differentiation [48]. cGMP-PKG signaling pathway is highly correlated with chondrocyte proliferation and differentiation and is involved in the development process of osteoarthritis [49]. Furthermore, both cell cycle and calcium signaling pathways play pivotal role in regulating chondrogenesis, chondrocyte homeostasis and cartilage repair [50, 51]. These results suggest that GZZSZTW treatment control multiple signaling pathways involved in chondrogenesis, chondrocyte proliferation and differentiation, and cartilage repair.

According to the protein–protein interaction analysis, TGFB1, RHOA, ILK, FLNA, RAC1, LYN and CDC42 were located in the center of the network and served as a hub to interact with other proteins. TGFB1 is a member of the TGFB family, which play critical roles in regulating chondrocyte differentiation from early to terminal stages, including condensation, proliferation, terminal differentiation, and maintenance of articular chondrocytes [52]. RHOA, RAC1 and CDC42 are three members of the RHO GTPase family, which play critical roles in governing numerous aspects of cell biology through a multitude of effecter pathways and proteins, including the organization of the actin cytoskeleton, cell polarity, cell cycle progression, membrane transport and transcription factor activity [53]. Many studies have shown that TGFB1 and its signaling pathway crosstalk with those RHO GTPases play pivotal roles in regulating chondrogenesis and cartilage regeneration [54–56]. FLNA is an actin binding protein that serves as an important upstream modulator of RHOA activation to effect normal chondrocytes development. In addition, FLNA can bind other small RHO GTPases such as RAC1 and CDC42 [57]. LYN is a member of the Src family kinases, which are important signaling intermediaries to regulate various cellular processes, such as proliferation, differentiation, apoptosis, migration and metabolism [58]. FGB and FGG are two isoforms of the fibrinogen molecules, which play overlapping roles in blood clotting, fibrinolysis, cellular and matrix interactions, inflammatory response and wound healing [59]. It has been shown fibrinogens can be activated to form a fibrin matrix to fill cartilage lesions and considered to be potentially effective for cartilage repair [60]. ILK is a crucial enzyme involved with integrin-mediated signal transduction in chondrocytes. Chondrocyte-specific ILK-inactivated mice develop chondrodysplasia and die at birth due to respiratory distress. The chondrodysplasia was characterized by abnormal chondrocyte shape, decreased chondrocyte proliferation with adhesion defects, failed spreading and fewer actin stress fibers, which indicates ILK plays pivotal role in cartilage growth and development [61]. The siRNA assay indicated that TGFB1 silencing in primary chondrocytes significantly decreased the expression levels of RHOA, ILK, CDC42, LYN and FLNA, and subsequently antagonized the effect of GZZSZTW-medicated serum on chondrocyte proliferation.

In addition to the above proteins located in the center of the interaction network, 5 proteins including DHX15, PKM, RAB15, RAB1B and GIPC1 were identified to interact with the above central proteins. However, there is still no obvious evidence regarding their roles in regulating cartilage growth and development. DHX15 is a DEAH-box helicase involved in RNA processing, splicing, and ribosome biogenesis, which impacts gene expression and cell proliferation [62]. PKM is an enzyme responsible for the conversion of pyruvate and ATP in glycolysis, and plays a key role in regulating cell metabolism and proliferation [63]. RAB15 and RAB1B are members of the RAB protein family, which regulate membrane trafficking, cell growth and differentiation [64]. GIPC1 is a cytoplasmic scaffold protein that is involved in cell proliferation, apoptosis, cell motility and adhesion [65]. Therefore, our results suggest that the therapeutic effects of GZZSZTW on joint diseases might be achieved through the TGFB1/RHO interaction network, which coordinately interact with multiple proteins and signaling pathways responsible for cartilage development, growth and repair.

## Conclusions

This study showed that the traditional Chinese herbal formula GZZSZTW controlled multiple proteins and signaling pathways responsible for the development, growth and repair of cartilage. GZZSZTW treatment might affect cell surface dynamics by regulating a series of serum proteins responsible for molecular connections between the plasma membrane and the cytoskeleton. GZZSZTW treatment might also control chondrogenesis, chondrocyte proliferation and differentiation and cartilage repair by modulating several signaling pathways, including hippo signaling pathway, cGMP-PKG signaling pathway, cell cycle and calcium signaling pathway. Finally, we identified an interaction network formed by TGFB1 and RHO GTPases together with several other proteins such as ILK, FLNA, LYN, DHX15, PKM, RAB15, RAB1B and GIPC1 by protein–protein interaction analysis and siRNA knockdown assay. Taken together, our results suggest that the effects of GZZSZTW on treating joint diseases might be achieved through the TGFB1/RHO interaction network coupled with other proteins and signaling pathways that were identified in this study. Therefore, the present study has greatly expanded our knowledge and provided scientific support for the underlying therapeutic mechanisms of GZZSZTW on treating joint diseases. It also provided possible alternative strategies for the prevention and treatment for joint diseases by using traditional Chinese herbal formulas.

## Data Availability

The datasets used and/or analyzed during the current study are available from the corresponding author on reasonable request.

## Abbreviations

GZZSZTW: Guzhi Zengsheng Zhitongwan
iTRAQ: isobaric tags for relative and absolute quantitation
PRM: parallel reaction monitoring
TGFB1: transforming growth factor beta-1
ILK: integrin-linked protein kinase
FLNA: filamin-A
RAB15: Ras-related protein Rab-15
RAB1B: Ras-related protein Rab-1B
